# Disrupted in schizophrenia 1 (*DISC1*) L100P mutants have impaired activity-dependent plasticity *in vivo* and *in vitro*

**DOI:** 10.1038/tp.2015.206

**Published:** 2016-01-12

**Authors:** D Tropea, I Molinos, E Petit, S Bellini, I Nagakura, C O'Tuathaigh, L Schorova, K J Mitchell, J Waddington, M Sur, M Gill, A P Corvin

**Affiliations:** 1 Neuropsychiatric Genetics Research Group, Department of Psychiatry, Trinity College Dublin, Dublin, Ireland; 2 Trinity College Institute of Neuroscience, Trinity College Dublin, Dublin, Ireland; 3 Department of Brain and Cognitive Science, Massachusetts Institute of Technology, Cambridge, MA, USA; 4 Department of Molecular and Cellular Therapeutics, Royal College of Surgeon in Ireland, Dublin, Ireland; 5 Smurfit Institute and Trinity College Institute of Neuroscience, Trinity College Dublin, Dublin, Ireland

## Abstract

Major neuropsychiatric disorders are genetically complex but share overlapping etiology. Mice mutant for rare, highly penetrant risk variants can be useful in dissecting the molecular mechanisms involved. The gene disrupted in schizophrenia 1 (*DISC1*) has been associated with increased risk for neuropsychiatric conditions. Mice mutant for *Disc1* display morphological, functional and behavioral deficits that are consistent with impairments observed across these disorders. Here we report that *Disc1* L100P mutants are less able to reorganize cortical circuitry in response to stimulation *in vivo*. Molecular analysis reveals that the mutants have a reduced expression of PSD95 and pCREB in visual cortex and fail to adjust expression of such markers in response to altered stimulation. *In vitro* analysis shows that mutants have impaired functional reorganization of cortical neurons in response to selected forms of neuronal stimulation, but there is no altered basal expression of synaptic markers. These findings suggest that *DISC1* has a critical role in the reorganization of cortical plasticity and that this phenotype becomes evident only under challenge, even at early postnatal stages. This result may represent an important etiological mechanism in the emergence of neuropsychiatric disorders.

## Introduction

The major neuropsychiatric disorders (for example, schizophrenia, bipolar disorder, major depressive disorder, autism spectrum disorder and attention deficit hyperactivity disorder) are substantially heritable and are of genetically complex etiology. Their etiology is polygenic, with evidence for a spectrum from common but small effects to rare, more highly penetrant mutations, together with environmental risk factors.^1^ Although age at onset varies across these disorders, from childhood (autism spectrum disorder, attention deficit hyperactivity disorder) to young adulthood (most cases of schizophrenia, major depressive disorder, bipolar disorder), they share not only overlapping symptoms and environmental risk factors, but also molecular etiology.^2^ Recent findings suggest that dysregulation of synaptic function and plasticity in cortex and hippocampus are related to cellular and behavioral alterations observed in neuropsychiatric disorders.^3^ Here, we show that a putative risk gene, disrupted in schizophrenia 1 (*DISC1*), implicated across neuropsychiatric disorders regulates not only the initial development of neuronal circuitry, but also synaptic plasticity during postnatal development *in vivo*. This role in activity-dependent reorganization of neural circuitry may explain how risk genes contribute to a range of phenotypic outcomes influenced by different environmental stimuli.

There is broad agreement that studying rare, highly penetrant risk mutations in appropriate cellular and animal models can shed light on the molecular mechanisms and neuronal circuit dysregulation contributing to neuropsychiatric disorders.^4^ *DISC1* was originally identified at the breakpoint of a balanced chromosomal translocation co-segregating with mental disorders in a large Scottish kindred.^5^ The strongest evidence indicated a broad-risk phenotype that includes schizophrenia and a range of neuropsychiatric disorders,^6, 7^ but the interpretation of these data has proved more challenging.^8, 9^ This gene, and in particular the initially reported mutation, has been the subject of much functional work.^10^ Systematic investigation suggests that DISC1 is present in several cellular compartments, including synapses, where it interacts with a wider molecular network to mediate cellular and synaptic function.^10, 11^ Recent studies on patient-derived iPSCs with a mutation in *DISC1* confirmed a cellular phenotype consistent with synaptic dysregulation.^12^ Disruption of DISC1's molecular interactions induces cellular phenotypes similar to those observed in neuropsychiatric disorders, hence specific DISC1-cofactor interactions may be a target for the treatment of mental disorders.^13^ One of the proven interactions of DISC1 is with phosphodiesterase 4B (PDE4B) and GSK3β, which also are risk factors for schizophrenia.^14^ Mouse models that disrupt these interactions (L100P mice) have been generated and show impairment in working memory^15^ and altered exploratory and habituation behavior,^16^ as well as deficits in brain size and neuronal migration.^17^ The DISC1–PDE4B interaction modulates the metabolism of cAMP, which is a second messenger important in neuronal signal transduction. cAMP-activated signaling determines the phosphorylation of CREB, which acts as a transcription factor for genes involved in neuronal and synaptic function. Indeed, CREB signaling is an important mediator of activity-dependent plasticity in health and disease.^18^ GSK3β controls molecular pathways dysregulated in several neuropsychiatric disorders,^19^ and in particular psychosis.^20^ Although there is evidence for involvement of DISC1 in synaptic transmission in several brain areas,^21, 22^ functional *in vivo* validation in response to long-term changes is still lacking. Here, we evaluate the ability of *Disc1* mutant mice to undergo activity-dependent reorganization of circuitry *in vitro* and *in vivo*. For the *in vivo* analysis, we use ocular dominance plasticity, an established paradigm to investigate reorganization of circuits in health and disease.^23^ For the *in vitro* analysis, we use primary neuronal cultures derived from homozygous L100P mutants and wild-type (WT) mice, and measure the expression of synaptic markers under basal conditions and after stimulation. We report that *in vivo* L100P mice fail to reorganize cortical circuits in response to long-term stimuli and that *in vitro* L100P cells have an impaired regulation of synaptic proteins in response to activity. In addition, we show that functional alteration is because of a dysregulation of CREB activity consequent to stimulation.

## Materials and methods

Animal studies were performed according to DCM regulations in each country (TCD-Ireland—license B100/4358 and MIT-USA). The DISC1 L100P mutants were provided by the RIKEN BRC through the National Bio-Resource Project of the MEXT, Japan. For preparation and immunocytochemistry, we used the methods previously described.^24^ Our primary neuronal cultures include neurons and glial cells, and 20% of neurons are gabaergic (Supplementary Figure 1). Disc1 is expressed in WT and L100P cultures and does not show a trend in specific neuronal type. For functional imaging and immunohistochemistry, we used the methods described in our past publications.^25, 26^ For detailed protocols *in vitro* and *in vivo*, see Supplementary Information.

### Choice of DISC1 mutant and colony management

As *DISC1* has proven to be a candidate gene for neuropsychiatric disorders, several mouse mutants have been generated with different alterations in the gene. Considering the multiple interactors of DISC1, it is reasonable to expect that different mutants will have different molecular and behavioral phenotypes, according to the pathway affected.^14^ The mice were maintained on a C57BL6 background (eight backcrosses).

The ENU-generated mouse of our choice has a single nucleotide mutation that affects the binding region of Disc1 with Pde4b and Gsk3β, which are two molecules involved in the pathology of schizophrenia. A recent work of Arime *et al.*^27^ shows that these mice have multiple ENU-induced mutations and that they sum up to produce the phenotype. Several studies characterized the behavioral phenotype of these mice. They demonstrate several phenotypes relevant to schizophrenia including impaired social behavior^27^ and hyperactivity in a novel environment.^16, 28^ Specifically, Walsh *et al.*^16^ replicated^16^ the hyperexploratory phenotype first reported by Clapcote *et al.*^15^ As reported by other authors,^28^ they failed to replicate the prepulse inhibition deficits reported for DISC1 L100P mutants in the original^15^ paper. Several studies also report morphological differences, such as reduced brain volume.^15^ It is of relevance that L100P mice do not have significant differences in Disc1 protein expression, but they show reduced expression of its interactors.^14, 27^ Consistent with these findings, Walsh *et al.*^16^ isolated Disc1 from WT and L100P striatum lysates and found that the interaction with Gsk3β was significantly reduced by the L100P mutation in our study. Altogether, these results show that L100P mice have molecular, behavioral and morphological deficits that are consistent with alterations observed in schizophrenia and other neuropsychiatric disorders, and that they are a valid model to study the biological function of Disc1 in such conditions.

## Results

### L100P mutants show impaired ability to respond to changes in neuronal activity *in vivo*

There is evidence that DISC1 is involved in synaptic function.^10^ To determine whether brain circuitry in L100P mice properly responds to neuronal stimulation *in vivo,* we tested the ability of these mutants to respond to altered sensory stimulation in visual cortex using the Ocular Dominance Plasticity paradigm. We first confirmed that the mice have an intact visual system and respond to visual stimulation, using the visual cliff test (Supplementary Figure 2). We evaluated, using optical imaging of intrinsic signals, the extent of cortical activity and map organization in L100P mutants versus controls. We find that, compared with age-matched controls, mutants do not show any impairment in cortical activation (WT Δ*R*/*R*=4.2±0.5 × 10−3; L100P Δ*R*/*R*=5.3±0.5 × 10−3) or in the formation of cortical maps (WT scatter =0.13±0.01; L100P Δ*R*/*R*=0.097±0.001).

**Figure 1:**
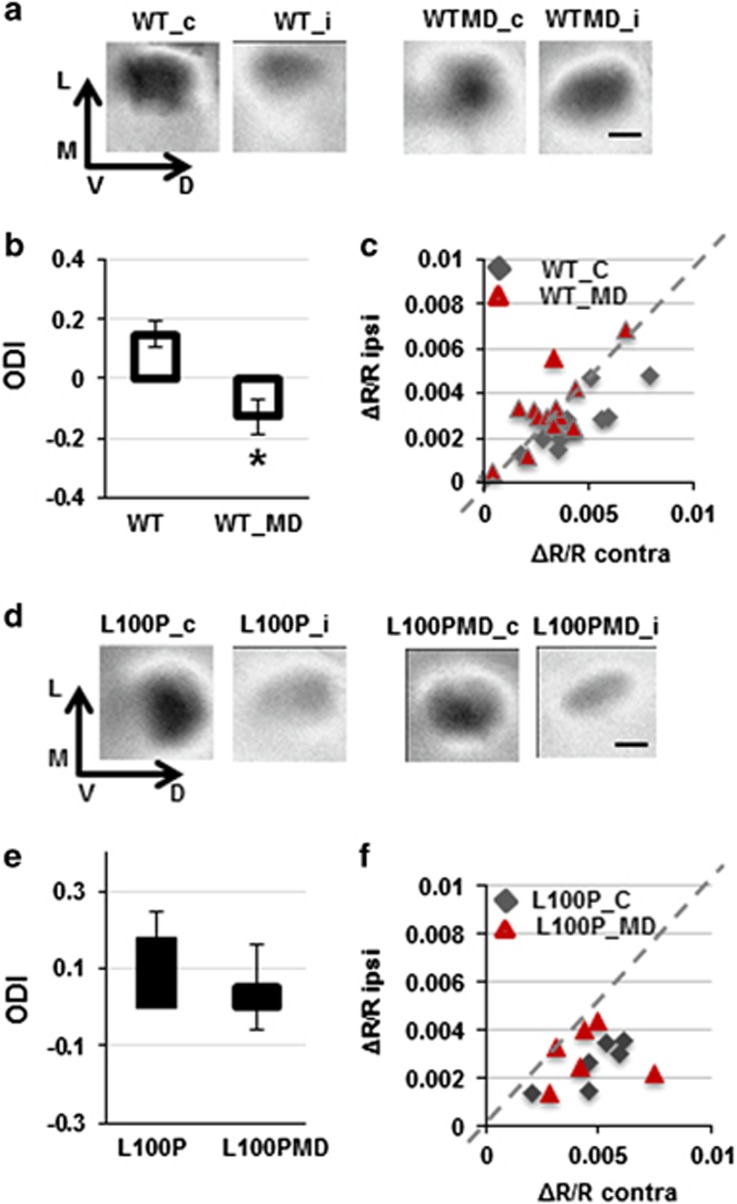
L100P mice have altered response to changes in sensory deprivation. Monocular deprivation induces a significant shift in ocular dominance index (ODI) in WT mice (**a**–**c**) but not in L100P mice (**d**–**f**). Plots of ocular dominance index in WT (**b**) and L100P mice (**e**) represent the overall contribution of both eyes in the reorganization of the circuitry; for details, see Supplementary Figure 1. (**c** and **f**) These panels report the individual contribution of contralateral (contra) and ipsilateral (ipsi) eyes in control (diamonds) and monocularly deprived (MD—triangles) mice, each point has the coordinates of contralateral eye (x) and ipsilateral eye (y). The ocular dominance shift after MD is visible in WT (**c**), but not in L100P mice (**f**). *Indicates statistically significant. Scale bar, 0.5 mm. WT, wild type.

We then tested how L100P mutants respond to sensory stimulation by measuring the ability of visual circuitry to reorganize in response to sensory deprivation using the ocular dominance index (ODI; Supplementary Figure 3). We find that during the critical period, deprivation of the dominant eye (monocular deprivation (MD)) induces the expected change in ODI in WT (ODI_WT_C=0.15±0.04, *N*=9; ODI_WT_MD=−0.13±0.06, *N*=12; Mann–Whitney (MW) =0.0014). However, the same protocol does not elicit any significant change in L100P mutants (ODI_L100P_C=0.18±0.07, *N*=6; ODI_L100P_MD=0.03±0.25, *N*=6; MW=0.13; Figure 1), showing that, although L100P mutants retain normal basal visual abilities, they do not manifest the significant shift in ocular dominance evident in WT mice. These results show that L100P mutants are impaired in responding to changes in sensory stimulation *in vivo*.

### Molecular pathways mediating activity-dependent response are altered in L100P mutant mice *in vivo*

**Figure 2:**
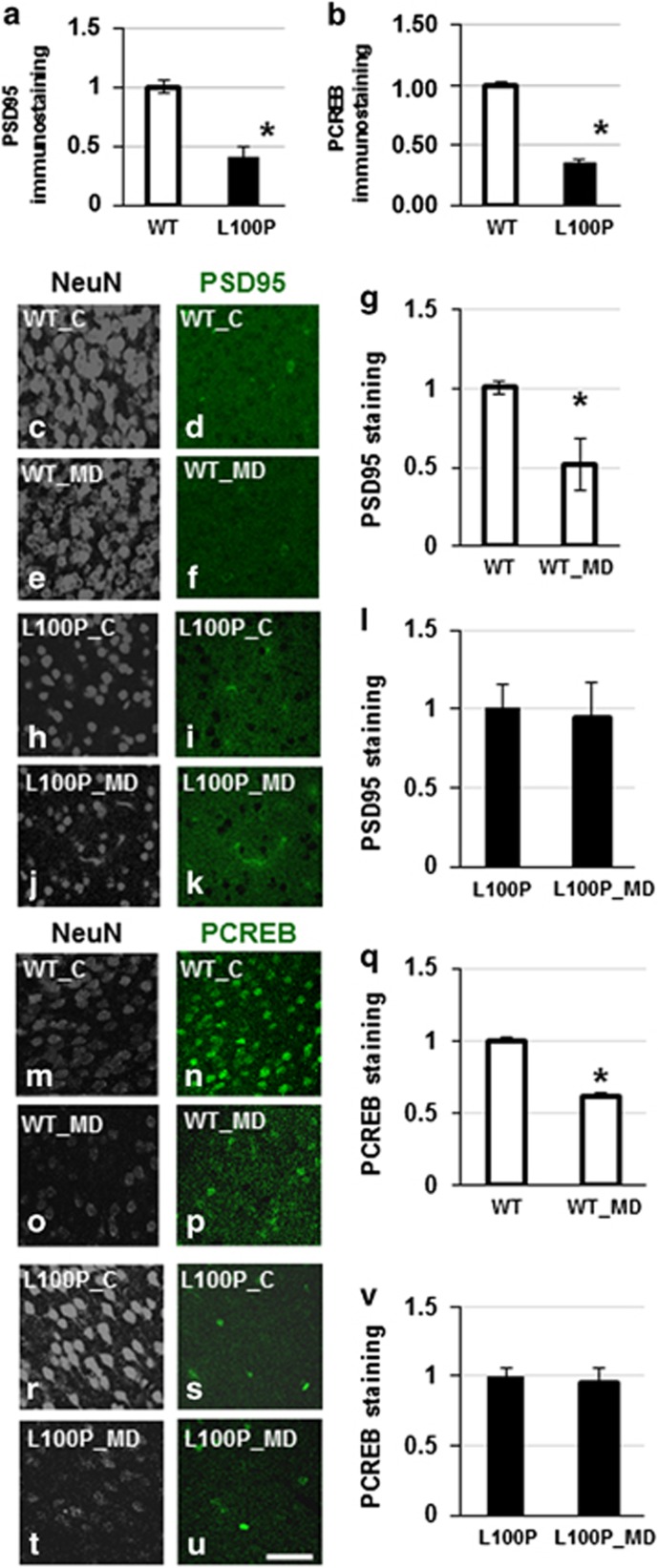
PSD95 and PCREB expression levels in WT L100P mice. (**a**) Measurements of PSD95 immunostaining in visual cortex sections of WT and L100P mice: the expression levels are significantly lower in the mutant. (**b**) The density of PCREB immunostaining significantly increases in WT but not L100P in visual cortex sections. (**c**–**v**) Immunostaining for PSD95 (**c**–**l**) shows a significant reduction of PSD95 expression in the deprived region of the visual cortex of WT (**g**), but not L100P (**l**) mice after monocular deprivation (MD). Similarly, PCREB expression (**m**–**v**) is significantly decreased in WT (**q**) but not L100P mice (**v**). Scale bar (**g**–**j**), 80 μm. *Indicates statistically significant. WT, wild type.

To establish the molecular mechanisms that underlie the inability of circuits to reorganize in response to MD, we performed immunohistochemistry on visual cortical sections where ocular dominance plasticity had been induced. We (1) measured the basal expression levels of proteins involved in synaptic function and activation of PCREB signaling, and (2) investigated protein levels in response to deprivation (Figure 2). We reasoned that the most significant changes should be observed in the contralateral visual cortex, as this area receives mostly inputs from the deprived eye.

**Figure 3:**
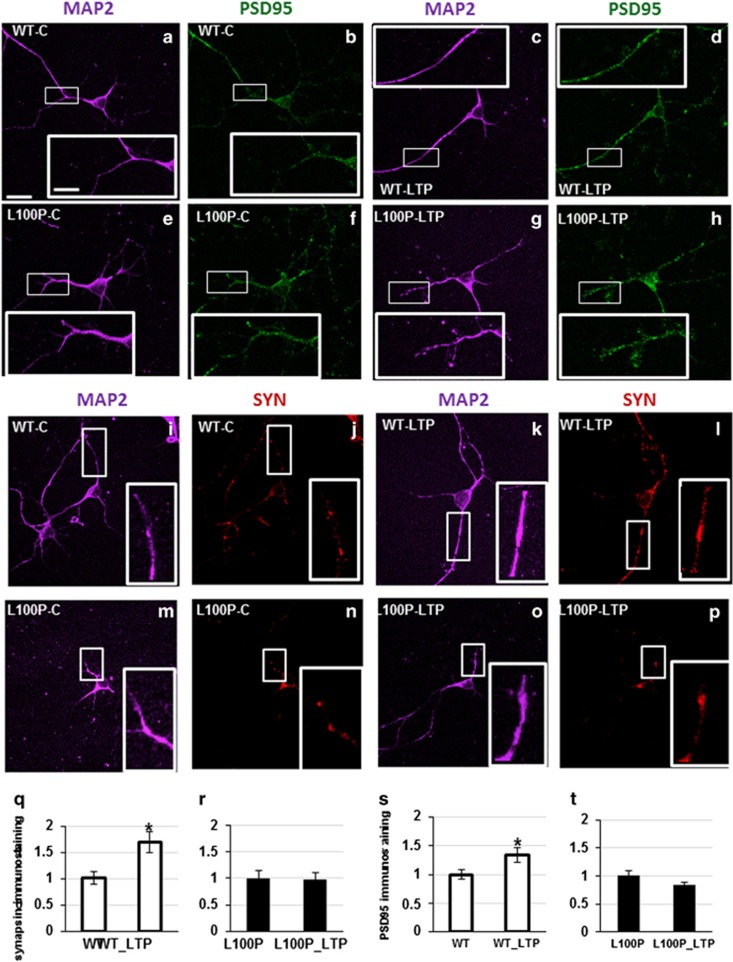
L100P mice do not show changes in PSD95 and synapsin expression after neuronal stimulation. Immunostaining for PSD95 and MAP2 (**a**–**h, s** and **t**) show a significant increase of PSD95 expression in the dendrites of primary neuronal cultures from WT, but not L100P mice after long-term potentiation (LTP) stimulation. Similarly, synapsin (SYN) expression is significantly increased in WT (**i**–**l**, **q**) but not L100P mice (**m**–**p, r**) after stimulation. Scale bar figures, 40 μm; scale bar blow-out, 10 μm. *Indicates statistically significant. WT, wild type.

First, we examined the expression of PSD95 and synapsin (SYN). We find that there is a significant (60%) reduction in basal levels of PSD95 immunostaining in the visual cortex of mutant mice compared with controls (PSD95_WT_control (*n*=9; *N*=3) average =1.00±0.05; PSD95_L100P_control (*n*=10; *N*=3) average =0.42±0.08; MW=0.0003; Figure 2). By inducing ocular dominance plasticity, we find, as expected, a decrease (30%) in PSD95 immunostaining in the contralateral visual cortex of WT (PSD95_WT_control (*n*=10; *N*=3) average =1.00±0.04; PSD95_WT_MD (*n*=5; *N*=3) average =0.52±0.16; MW=0.01; Figure 3a–f). This result is driven by a significant reduction in staining from the monocular portion of the contralateral cortex, which receives inputs from the deprived eye only (Figure 2, Supplementary Figure 3; 50% MW=0.01). In mutants, there are no significant changes in PSD95 immunostaining in response to ocular dominance plasticity, even when we consider only the areas that receive input exclusively from the deprived eye PSD95_L100P_control (*n*=8; *N*=3) average =1.00±0.15; PSD95_L100P_MD (*n*=7; *N*=3) average =0.94±0.23; MW=NS (not significant).

Although the expression of PSD95 immunostaining is altered, we do not find any significant change in the expression levels of SYN in WT versus L100P mutant mice and in the response to MD, in either genotype, even considering the monocular area of the deprived section. The unaltered expression of SYN suggests that there are mechanisms which counterbalance activity-related changes at the level of excitatory postsynaptic structures, so that the total number of connections is not altered.

As other L100P mutants have altered PCREB expression,^29^ and CREB phosphorylation is important for visual cortical plasticity, we checked whether impairment in CREB activation is present in visual cortical sections and if the changes in CREB activation induced by MD are consistent in WT and mutant animals. The basal expression of PCREB in the visual cortex is reduced (70%) in L100P mutants compared with the control levels (PCREB_WT (*n*=244, *N*=3) average =1.00±0.02 (s.e.), PCREB_L100P (*n*=135, *N*=3) average =0.35±0.03, MW=0.0001; Figure 2b). MD induces a significant reduction in PCREB immunostaining in WT visual cortex (WT_control (*n*=375; *N*=3) average = 1.00±0.02 (s.e.); WT_MD (*n*=178; *N*=3) average 0.62±0.02 (s.e.), MW=0.0001, Figure 2). The same protocol does not induce any change in the contralateral cortex of the mutants (L100P_control (*n*=135; *N*=3) average =1.00±0.06; L100P_MD (*n*=61; *N*=3) average =0.95±0.10. MW=NS; Figure 2). Note that although MD does not lead to any significant change with respect to basal levels of the mutants, PCREB immunostaining is still significantly lower (30%) with respect to the level observed in the deprived cortex of WT (WT_MD_contralateral =0.80±0.04; mut_MD_contralateral =0.50± 0.03; MW=0.0001). These results show that L100P mutants have abnormal activation of CREB signaling in response to altered sensory stimulation *in vivo*.

### L100P mutant neurons show impaired reactivity to neuronal stimulation *in vitro*

Our data *in vivo* show that L100P mutants are unable to respond properly to neuronal stimulation. To study synaptic plasticity at early stages of postnatal development, we prepared primary cellular cultures from the cortex of L100P mutants and WT littermates at P0–P1. We challenged the cultures with long-term potentiation (LTP) and long-term depression (LTD), to reproduce the phenomena observed *in vivo*, using the ocular dominance plasticity paradigm.^30^ We observe no significant differences in the basal immunostaining of presynaptic (SYN) and postsynaptic (PSD95) markers between DISC1 mutants and WT littermates (Supplementary Figure 4, Table 1). However, when we stimulated the cells for LTP, the cultures derived from these two genotypes reacted differently. Stimulation elicited a significant (25%) increase in immunostaining of both PSD95 and SYN in WT, but no changes in protein expression are present in L100P mutant mice. Detailed results are reported in Table 1 and showed in Figure 3.

We tested whether the increase in expression of synaptic markers in WT is because of an increase in the number or in the size of the synapses. Using particle analysis of PSD95 immunostaining, we find that the increase is because of enrichment in the number of puncta, rather than an increase in synapse size (Supplementary Figure 4, Table 2), suggesting that LTP stimulation induces an increase in the number of excitatory synapse in WT but not in mutants. No changes were observed in the pattern of SYN puncta, *P*-value MW=0.54. At the basal level, there is no difference in the PSD95 and SYN puncta between WT and L100P (Table 2).

To analyze the overlapping of presynaptic and postsynaptic markers and determine the presence of synapses, we analyzed the overlapping of the two different fluorophores (red for SYN and green for PSD95) and we compared the amount of yellow staining (overlapping areas) in WT versus L100P. The amount of overlapping should correlate with the number of synapses. We did not find a significant difference in the amount of functional synapses between WT and mutants, although the L100P show a reduced overlapping. (Colocalization number: WT-basal =0.46±0.02, WT-LTP =0.47±0.02, *n*=16 cells, *N*=3 animals; L100P-basal =0.35±0.03, L100-LTP =0.36±0.03, *n*=14 cells, *N*=3 animals; *P*-value =0.5; WT-basal =0.5±0.02, WT-LTD =0.4±0.04, *n*=16 cells, *N*=3 animals; L100P-basal =0.5±0.02, L100-LTD =0.5±0.02, *n*=16 cells, *N*=3 animals).

**Figure 4:**
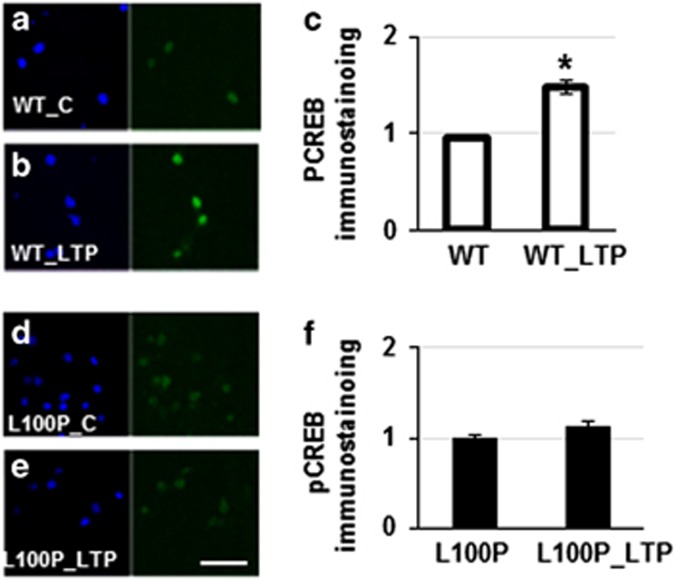
Activation of CREB in response to stimulation is impaired in L100P mice *in vitro*. (**a**–**c**) Neuronal stimulation in cultures derived from WT animals lead to a significant increase in the expression of activated creb (PCREB), whereas no effect is present in cultures derived from L100P mice (**d**–**f**). *Indicates statistically significant. Scale bar, 40 μm. LTP, long-term potentiation; WT, wild type.

We then investigated the molecular mechanisms underlying the inability of L100P mutants to react to stimulation. According to the literature,^15^ these mutants have a decreased ability to bind Pde4B and this impacts on the metabolism of cAMP. As cAMP is an important modulator of activity-dependent cellular response and mediates CREB-dependent activation of transcription,^31^ we reasoned that in mutant-derived neurons there should be an altered reduced concentration of PCREB, which corresponds to the activated form of CREB. Indeed, we find that after 8 days *in vitro* in regular culture conditions, L100P mutants evidence 50% less PCREB immunostaining compared with WT: PCREB_WT (*n*=159, *N*=2) average =1.00±0.03, PCREB_L100P (*n*=110, *N*=2) average =0.46±0.02 (Supplementary Figure 5). Neuronal stimulation induces significant CREB activation in WT, but not in L100P mice, suggesting that these mutants are impaired in responding to stimulation via CREB activation WT_control (*n*=204; *N*=3) average =1.00±0.03; WT_LTP (*n*=142; *N*=3) average =1.48±0.07; WT_LTP L100P_control (*n*=199; *N*=5) average =1.00±0.02; WT_LTP L100P_LTP (*n*=68; *N*=5) average =1.00±0.10 (Figure 4).

Interestingly, another form of stimulation: LTD did not cause any change in any of the markers tested (Table 3). At a later time in cultures (15–20 days *in vitro*), we did not observe similar phenotype, suggesting that this deficit is evident at the time of synapses' formation.

Altogether, our analysis *in vitro* and *in vivo* shows that L100P mutants have an abnormal response to neuronal stimulation, and that impairment at the synaptic level is correlated with reduced PCREB, and as a consequence, decreased PCREB-mediated signaling.

### L100P mutants have altered levels of DISC1-interactors

**Figure 5:**
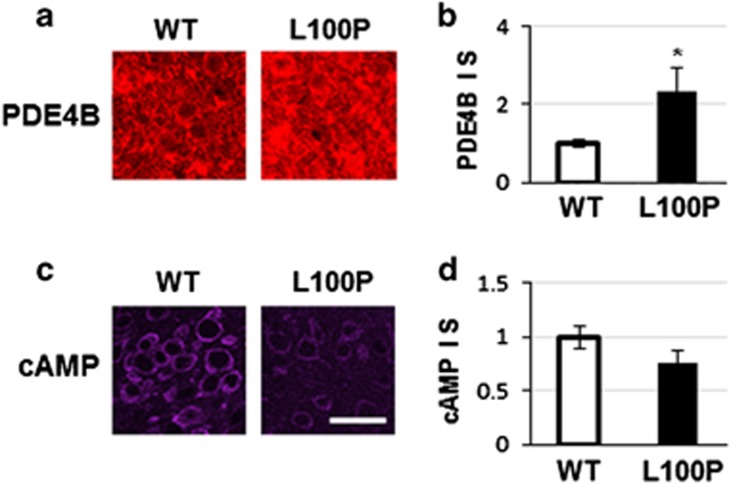
Alteration of PDE4B pathway in L100P mice. (**a**) PDE4B immunostaining is increased in the brain of L100P mice. Representative images from PFC. (**b**) Quantification of PDE4B staining across different brain regions shows significant increase of PDE4B expression in L100P mice. (**c**) cAMP immunostaining is decreased in the brain of L100P mice. Representative images from PFC. (**d**) Quantification of cAMP staining across different brain regions shows a decreased trend of cAMP expression in L100P mice. Scale bar, 40 μm. *Indicates statistically significant. PFC, prefrontal cortex; WT, wild type.

In an attempt to clarify the molecular mechanisms that lead to the unpaired activity-dependent plasticity in L100P mutants, we performed immunohistochemistry for Pde4B and cAMP in adult L100P mice in different brain areas: prefrontal cortex, visual cortex, hippocampus and cerebellum. We find an overall significant increase of immunostaining in Pde4B and a nonsignificant trend for reduced cAMP staining (Figure 5).

As the specific mutation should impact cAMP metabolism and ultimately the activation of CREB signaling, we measured the levels of CREB and PCREB in nuclear fractions from L100P mutant and WT brains. We found comparable levels of both proteins between mutants and WT in whole-brain extracts and across different brain sections, however, there is a decreased CREB and PCREB immunostaining in the prefrontal cortex, (*N*=2 animals, *n*=4 sections, CREB WT =1±0.16, L100P=m=0.57±0.18; PCREB WT =1±0.12, L100P=0.55±0.12) an area directly involved in working memory and neuropsychiatric conditions. In addition, we performed quantification of Gsk3β in whole-brain extracts, and we found a significant reduction of Gsk3β immunostaining in adults (WT=1±0.02; L100P=0.2±0.05; *N*=6 animals, *P*-value =0.01; Supplementary Figure 6). We confirmed a reduced immunostaining for Gsk3β also in brain extracts immunoprecipitated for Disc1 (Walsh *et al.*, 2012, unpublished data), suggesting that the overall decrease in Gsk3β can be either due to a reduced overall concentration of the protein or to a reduced interaction with Disc1.^20^ In summary, the mutation in Disc1 protein, determines changes in Disc1-mediated pathway and in the concentration of Disc1 interactors Pde4B and Gsk3β.

### L100P mutants have altered expression of cellular and synaptic markers during development

In an attempt to clarify the developmental expression of synaptic markers *in vivo*, we performed protein expression analysis in brain extracts from WT and L100P mice at different ages: synaptic formation (P8–10), synaptic pruning (P30–40), and adulthood (above P60). For the analysis of synaptic proteins, we isolated synaptoneurosomes from total brain extracts and we measured the expression of presynaptic (SYN) and postsynaptic (PSD95) markers. We find that the expression of SYN in WT versus L100P mutants has a trend which is dependent on the developmental stage: the expression at P8–10 SYN levels in mutants was more than twofold higher than in age-matched controls (Supplementary Figure 7; P8–10 WT=1±0.095, L100P=1.4±0.13, *N*=12 animals, *P*-value =0.01), while at P30–40 the mutants show 35% reduction in SYN levels (P30 WT=1±0.1, L100P=0.62±0.2, *N*=12 animals, *P*-value =0.4) and more than 40% reduction in adulthood (P60 WT=1±0.03, L100P=0.58±0.08, *N*=12 animals, *P*-value =0.01). The expression of PSD95 follows a different developmental trend: at P8–10, there are no significant differences in the expression of PSD95 in WT and mutants (although there is a 20% increase in mutants versus WT: P30 WT=1±0.9, L100P=1.2±0.1, *N*=9 animals, *P*-value =0.4). Expression of PSD95 is significantly reduced in mutants versus WT at P30–40 (WT=1±0.06, L100P=0.7±0.7, *N*=9 animals, *P*-value =0.01) while P60 brains express comparable levels of the protein (WT=1±0.12, L100P=0.96±0.11, *N*=12 animals; Supplementary Figure 7).

In summary, we find that the expression pattern of synaptic proteins changes during development, with an unexpected increase in SYN in mutants versus WT at P8–10, and a progressive decrease at the following developmental stages.

As the specific mutation should impact cAMP metabolism and ultimately the activation of CREB signaling, we measured the levels of CREB and PCREB in nuclear fractions from L100P mutant and WT brains. We found comparable levels of both proteins between mutants and WT in whole-brain extracts at all postnatal ages. In the same preparation, we confirmed a significant reduction of Gsk3β immunostaining in adults (WT=1±0.02; L100P=0.2±0.05; *N*=6 animals, *P*-value =0.01). In summary, the activation of cellular pathways between WT and L100P mutants decreases over development.

## Discussion

The main outcome of this study is that stimulation challenges reveal deficiencies in mutants for risk genes of neuropsychiatric disorders, even at an early stage of postnatal development, and the impairments become evident only in response to a specific challenge.

### Highly penetrant genetic mutations are models of choice for the study of neuropsychiatric conditions

We choose to study *DISC1* for a number of reasons. First, a translocation interrupting this gene is one of the first identified high penetrance mutations for common psychiatric outcomes.^5^ Second, other variants at this gene have been implicated across neuropsychiatric disorders.^13^ Third, physical interactors of DISC1 have also been implicated in these disorders, suggesting that the molecular mechanism modulated by DISC1 may be critical across a number of disorders.^7, 14, 32^ There are several models of *Disc1* mutants,^29^ and we decided to use L100P mutants, which present a selective disruption of Disc1 interaction with selected molecules, such as Pde4b and Gsk3β, which are also potential risk genes for schizophrenia.^20^ We reasoned that as Pde4b is directly involved in the metabolism of cAMP, an important mediator of CREB pathway and activity-dependent development, these mutants may show impairment in the formation and remodeling of circuitry. The CREB pathway has been identified as an important modulator of activity-dependent plasticity and learning abilities in several organisms.^33^ Indeed we find a correlation between impaired activity-dependent changes and activation of CREB in L100P mice, both *in vitro* and *in vivo*. Reduced CREB activity may impact both intrinsic and evoked excitability of the circuits^33^ and affect the efficacy of the communication from synapse to nucleus to synapse communication^18^ (Supplementary Figure 8). There are several cellular systems that ultimately activate CREB,^31^ and the main effector is thought to be cAMP, whose concentration is regulated by PDE4B.

In our study, we assess the role of *Disc1* in activity-dependent reorganization of circuitry *in vivo*. In addition, we show that some deficits in circuitry reorganization can be observed *in vitro* even at early phases of postnatal development under appropriate conditions.

*In vivo*, basal expression of PSD95 is significantly downregulated in the visual cortex of juvenile L100P animals (Figure 2), consistent with the impairment of glutamatergic transmission reported in schizophrenia and other disorders;^12, 34^ this is confirmed by immunoblotting analysis of PSD95 on the whole brain. It is possible that the expression of markers for synaptic and cellular function changes according to brain region and developmental stage; in fact, the PSD95 reduction is not visible at younger ages (Supplementary Figure 7). After stimulation, L100P mutants fail to respond to molecular and physiological changes induced by activity in contrast to WT. Contrary to what was observed in basal conditions *in vivo*, we did not find any difference in control expression levels of synaptic markers between L100P mutants and WT in cultures (Supplementary Figure 4). This similarity in expression levels can be due to *in vitro* preparation, to the young age of the cultures (8–14 days *in vitro*) or to the brain area selected. However, *in vitro*, LTP stimulation induces an effect in the expression levels of synaptic markers in WT, but not L100P cultures, hence *Disc1* mutation is involved in the LTP-mediated response.

We did not find significant changes with LTD stimulation, suggesting that specific pathways are disrupted, at least in this preparation.

### *In vivo* and *in vitro* studies reveal overlapping phenotypes in response to stimulation

Cultures prove to be good models to uncover morphological and molecular abnormalities linked to *DISC1* loss of function,^12, 35, 36^ and have the potential to reveal additional phenotypes in response to stimulation protocols that cannot be performed *in vivo*. Our experiments on the properties of circuitry in L100P mutant preparations suggest a new interpretation of circuit function in neuropsychiatric disorders: even if there is no physiological difference in the basal conditions of the circuitry between mutants and WT, challenging the system can reveal distinct phenotype, suggesting that cultures still retain the machinery to respond to activity but the underlying mechanisms are altered in L100P mutants even at initial phases of development. This may be important in modeling the dynamic processes that are likely to contribute to the etiology of neuropsychiatric disorders, and has a potential application to several cellular preparations: from primary neuronal cultures in mice to neuronal cells derived from patients. Consistent with the results *in vitro*, the physiological status of L100P juvenile mice were not altered *in vivo*, as we did not detect any impairment in the visual ability or cortical-evoked signal; however, these mutant mice were not able to respond to MD in the manner of WT controls (Figure 1). To our knowledge, this is the first study showing that appropriate stimulation conditions can uncover cellular phenotypes, not evident under basal conditions, even at early stages of development and in such cellular preparations.

### Mediators of molecular and functional phenotype in DISC1 mutants

The past decades have identified many molecular effectors of neuropsychiatric disorders to be involved in synaptic function. This aspect is indeed crucial for the adaptation of the brain circuitry to the changing environment and it is therefore expected that this phenotype would be impaired in neuropsychiatric conditions. There are several levels at which the circuitry could fail in responding to the extracellular signals: (1) communication between synapse and nucleus, (2) reorganization of synapses, (3) control of gene expression. DISC1, with its numerous interactors and molecular functions, is possibly involved in all these steps. In particular, the L100P mutants have impaired interactions with Pde4B and Gsk3β, which have both shown to be involved in activity-dependent reorganization of the synapses. Pde4B modulates the interplay between Disc1 and CREB activation.

In our mutants, the Disc1 protein is unable to interact properly with Pde4B, leading to an increased degradation of cAMP, and as a consequence, the level of PCREB is decreased. Indeed histochemical analysis of Pde4B and cAMP levels in prefrontal cortex reinforce the finding that the cAMP–CREB pathway is disrupted (Figure 5). However, it is possible that the mechanisms leading to CREB phosphorylation are different in different brain areas, and that phosphodiesterases are also involved in the balance of CREB activation.^29, 37^ We cannot exclude that there could be different mechanisms that mediate the limited plasticity in L100P mice: Wei *et al.*^38^ report that *in vitro*, silencing *Disc1* causes an increase of NMDAR activation. The constant upregulation of activity may cause a homeostatic imbalance that impairs the ability of mutant cells to respond to changes of activity. Also, the role of Disc1 at the synapse has been reported by other studies that show the modulation of glutamatergic components via interactions with TNIK and Kalirin7;^39, 40^ however, in our mutants these are possibly indirect interactions as the mutated site in Disc1 protein is different from that affected in L100P mice.

Another possible mechanism that explains L100P dysfunction is the impairment of Gsk3B, with its consequences on PSD95 distribution. Gsk3β activating pathways are involved in the localization of PSD95 to synapses in response to neuronal stimulation.^41^ The localization of PSD95 at excitatory synapses is directly correlated to synaptic strength^42^ and has a functional consequence with the glutamatergic impairment in psychosis.^34^ The impairment of PSD95 but not SYN, which we find in our *in vivo* preparations, can be directly correlated to this pathway as a consequence of the disruption of Gsk3β pathway, and can be related to deficits in synapses strengthening, rather than formation. In fact, not only the presynaptic expression of synapsis is related to both excitatory and inhibitory synapses, pointing at a possible imbalance between gabaergic and glutamatergic transmission, but SYN puncta are also associated to newly formed synaptic boutons, which appear even before the postsynaptic elements are assembled and can disappear if the synapse is not strong enough to be maintained. These additional mechanisms will need to be analyzed in detail in the following-up studies, considering cell-specificity and developmental stage.

### The expression of molecular markers adapts through development to compensate the alteration in basal expression levels

Considering that the L100P system was shown to be less reactive to stimulation challenges right after birth and in juvenile animals, we examined the expression of cellular and synaptic markers during development in WT and L100P mice, finding a specific pattern for different markers: although PCREB expression does not change over time between WT and L100P, the finding that the overexpression of presynaptic markers at the time of synapse formation is followed by a progressive decay at following stages of development suggests that the system tends to adapt to the initial bias compensating the excess with a subsequent decrease at the time of pruning. A consequence of the deficits in presynaptic markers can be the deficit in presynaptic release observed in other *Disc1* mutants which also impact NMDA activation.^38, 43^ Even considering that the results may be different in specific brain areas, and that the information is lost in whole-brain homogenates, these results have profound implications that extend to the general biology of neuropsychiatryc disorders and deserve to be further studied considering other mutants.

We suggest that under normal developmental conditions, the system adjusts to the genetic mutation with compensatory mechanisms;^44^ however, when the system is challenged, these mechanisms do not suffice and mutants manifest an altered response. It is possible that the initial impairment will be rectified in the longer term by compensatory mechanisms. This explanation is consistent with the impaired short-term plasticity observed in other mice mutants for *Disc1.*^29^

### Plasticity for discovering hidden phenotypes in neuropsychiatric conditions

Interestingly, even before circuitry has reached maturation, stimuli used to induce circuitry reorganization may be used to uncover the phenotype. Of particular interest are *in vivo* forms of plasticity such as visual cortical plasticity. Even if patients with neuropsychiatric disorders do not have apparent deficits in visual function, there is growing evidence that visual perception is altered in schizophrenia,^45^ and visual system plasticity has been used in other neurodevelopmental disorders to uncover the biology of the disease and to test possible treatments.^46, 47^ Indeed, there is agreement that the visual system is a choice model for studying the reorganization of circuitry *in vivo*.^23^ These observations suggest that future studies comparing mutations of different severity, or assays representing variable mutation burden, may be useful for modeling neuropsychiatric phenotypes. Interestingly, we did not observe any phenotype in response to LTD *in vitro*, suggesting that the mechanisms evoked by LTP and LTD are different, and therefore only specific forms of stimulation will reveal the dysregulation induced by mutant genes, according to the mechanisms in which the genes are involved or the area of investigation.^48^

## Figures and Tables

**Table 1:** Basal level of expression of PSD95 and synapsin in WT and L100P animals

*Antigen*	n *(cells)*	N *(animals)*	*Immunostaining*	*s.e.*	n *(neurites)*	*Rel puncta*	*s.e.*
PSD95-WT	15	3	1	0.01	20	0.22	0.02
SYN-WT	15	3	1	0.01	20	0.07	0.01
PSD95-L100P	14	3	1.1	0.01	20	0.22	0.02
SYN-L100P	14	3	0.9	0.02	20	0.15	0.01

Abbreviations: SYN, synapsin; WT, wild type.

**Table 2:** Immunostaining and puncta analysis of LTP-treated and control cultures

*Genotype*	*Treatment*	*Antigen*	n *(cells)*	N *(animals)*	*Average*	*s.e.*	*Neurites*	*Rel puncta*	*s.e.*
WT	Control	PSD95	40	3	1	0.08	40	0.14	0.02
WT	LTP	PSD95	36	3	1.3	0.03	40	0.19	0.02
L100P	Control	PSD95	68	5	1	0.01	57	0.11	0.01
L100P	LTP	PSD95	68	5	0.83	0.05	57	0.11	0.01
WT	Control	SYN	40	3	1	0.1	37	0.12	0.01
WT	LTP	SYN	37	3	1.7	0.2	37	0.19	0.02
L100P	Control	SYN	68	5	1	0.03	64	0.15	0.02
L100P	LTP	SYN	68	5	1	0.02	68	0.14	0.01

Abbreviations: LTP, long-term potentiation; SYN, synapsin; WT, wild type.

**Table 3:** Immunostaining of LTD-treated and control cultures

*Genotype*	*Treatment*	*Antigen*	n *(cells)*	N *(animals)*	*Average*	*s.e.*
WT	Control	PSD95	28	3	1	0.07
WT	LTD	PSD95	28	3	1	0.01
L100P	Control	PSD95	20	5	1	0.06
L100P	LTD	PSD95	20	5	1	0.15
WT	Control	SYN	17	3	1	0.15
WT	LTD	SYN	17	3	0.95	0.2
L100P	Control	SYN	17	5	1	0.09
L100P	LTD	SYN	17	5	1.3	0.22

Abbreviations: LTD, long-term depression; SYN, synapsin; WT, wild type.

## References

[bib1] Sullivan PF, Daly MJ, O' Donovan M. Genetic architectures of psychiatric disorders: the emerging pictures and its implications. Nat Rev Genet 2012; 13: 537–551. 22777127 10.1038/nrg3240 PMC4110909

[bib2] Cross-Disorder Group of the Psychiatric Genomics Consortium. Genetic relationship between five psychiatric disorders estimated from genome-wide SNPs. Nat Genet 2013; 45: 984–994. 23933821 10.1038/ng.2711 PMC3800159

[bib3] Penzes P, Buonanno A, Passafaro M, Sala C, Sweet RA. Developmental vulnerability of synapses and circuits associated with neuropsychiatric disorders. J Neurochem 2013; 126: 165–182. 23574039 10.1111/jnc.12261 PMC3700683

[bib4] Karayiorgou M, Flint J, Gogos JA, Malenka RC, Genetic and Neural Complexity in Psychiatry 2011 Working group. The best of times, the worst of times for psychiatric diseases. Nat Neurosci 2012; 15: 811–812. 22627793 10.1038/nn.3115 PMC4416402

[bib5] Millar JK, Wilson-Annan JC, Anderson S, Christie S, Taylor MS, Semple CA et al. Disruption of two novel genes by a translocation co-segregating with schizophrenia. Hum Mol Genet 2000; 9: 1415–1423. 10814723 10.1093/hmg/9.9.1415

[bib6] Sachs NA, Sawa A, Holmes SE, Ross CA, DeLisi LE, Margolis RL. A frameshift mutation in Disrupted in Schizophrenia 1 in an American family with schizophrenia and schizoaffective disorder. Mol Psychiatry 2005; 10: 758–764. 15940305 10.1038/sj.mp.4001667

[bib7] Bradshaw NJ, Porteous DJ. DISC1-binding proteins in neural development, signaling and schizophrenia. Neuropharmacology 2012; 62: 1230–1241. 21195721 10.1016/j.neuropharm.2010.12.027 PMC3275753

[bib8] Sullivan PF. Questions about DISC1 as a genetic risk factor for schizophrenia. Mol Psychiatry 2013; 18: 1050–1052. 24056909 10.1038/mp.2012.182 PMC3967792

[bib9] Porteous DJ, Thomson PA, Millar JK, Evans KL, Hennah W, Soares DC et al. DISC1 as a genetic risk factor for schizophrenia and related major mental illness: response to Sullivan. Mol Psychiatry 2014; 19: 141–143. 24457522 10.1038/mp.2013.160 PMC4238281

[bib10] Brandon NJ, Sawa A. Linking neurodevelopmental and synaptic theories of mental illness through DISC1. Nat Rev Neurosci 2011; 12: 707–722. 22095064 10.1038/nrn3120 PMC3954824

[bib11] Camargo LM, Collura V, Rain JC, Mizuguchi K, Hermjakob H, Kerrien S et al. Disrupted in Schizophrenia 1 interactome: evidence for the close connectivity of risk genes and a potential synaptic basis for schizophrenia. Mol Psychiatry 2007; 12: 74–86. 17043677 10.1038/sj.mp.4001880

[bib12] Wen Z, Nguyen HN, Guo Z, Lalli MA, Wang X, Su Y et al. Synaptic dysregulation in human iPS cell model of mental disorder. Nature 2014; 20: 414–418. 10.1038/nature13716 PMC4501856 25132547

[bib13] Hikida T, Gamo NJ, Sawa A. DISC1 as a therapeutic target for mental illnesses. Expert Opin Ther Targets 2012; 16: 1151–1160. 23130881 10.1517/14728222.2012.719879 PMC3983783

[bib14] Lipina TV, Roder JC. Disrupted-In-Schizophrenia-1 (DISC1) interactome and mental disorders: impact of mouse models. Neurosci Biobehav Rev 2014; 45: 271–294. 25016072 10.1016/j.neubiorev.2014.07.001

[bib15] Clapcote SJ, Lipina TV, Millar JK, Mackie S, Christie S, Ogawa F et al. Behavioral phenotypes of *Disc1* missense mutations in mice. Neuron 2007; 54: 387–402. 17481393 10.1016/j.neuron.2007.04.015

[bib16] Walsh J, Desbonnet L, Clarke N, Waddinigton JL, O'Tuathaigh CM. Disruption of exploratory and habituation behavior in mice with mutation of DISC1: an ethologically based analysis. J Neurosci Res 2012; 90: 1445–1453. 22388794 10.1002/jnr.23024

[bib17] Lee FH, Fadel MP, Preston-Maher K, Cordes SP, Clapcote SJ, Price DJ et al. *Disc1* point mutations in mice affect development of the cerebral cortex. J Neurosci 2011; 31: 3197–3206. 21368031 10.1523/JNEUROSCI.4219-10.2011 PMC6623921

[bib18] Cohen S, Greenberg ME. Communication between the synapse and the nucleus in neuronal development, plasticity, and disease. Annu Rev Cell Dev Biol 2008; 24: 183–209. 18616423 10.1146/annurev.cellbio.24.110707.175235 PMC2709812

[bib19] Ayokunmi Ajetunmobi and Daniela TropeaProgress toward Therapies and Interventions for Neurodevelopmental DIsorders. Wiley ‘The Genetics of neurodevelopmental disorders': Singapore, 2015, pp 319–343.

[bib20] Lipina TV, Kaidanovich-Beilin O, Patel S, Wang M, Clapcote SJ, Liu F et al. Genetic and pharmacological evidence for schizophrenia-related Disc1 interaction with GSK-3. Synapse 2011; 65: 234–248. 20687111 10.1002/syn.20839 PMC4485461

[bib21] Kvajo M, McKellar H, Arguello PA, Drew LJ, Moore H, MacDermott AB et al. A mutation in mouse Disc1 that models a schizophrenia risk allele leads to specific alterations in neuronal architecture and cognition. Proc Natl Acad Sci USA 2008; 105: 7076–7081. 18458327 10.1073/pnas.0802615105 PMC2383956

[bib22] Holley SM, Wang EA, Cepeda C, Jentsch JD, Pletnikov MV, Levine MS. Frontal cortical synaptic communication is abnormal in *Disc1* genetic mouse models of schizophrenia. Schizophr Res 2013; 146: 264–272. 23481583 10.1016/j.schres.2013.02.007 PMC3622830

[bib23] Hofer SB, Mrsic-Flogel TD, Bonhoeffer T, Hubener M. Lifelong learning: ocular dominance plasticity in mouse visual cortex. Curr Opin Neurobiol 2006; 16: 451–459. 16837188 10.1016/j.conb.2006.06.007

[bib24] Corvin AP, Molinos I, Little G, Donohue G, Gill M, Morris D et al. IGF1 and its active peptide (1-3)IGF1 enhance the expression of synaptic markers in neuronal circuits through different cellular mechanisms. Neurosci Lett 2012; 520: 296–303. 10.1016/j.neulet.2012.05.029 22609570

[bib25] Tropea D, Kreiman G, Lyckman A, Mukherjee S, Yu H, Horng S et al. Gene expression changes and molecular pathways mediating activity-dependent plasticity in visual cortex. Nat Neurosci 2006; 9: 660–668. 16633343 10.1038/nn1689

[bib26] Tropea D, Majewska A, Garcia R, Sur M. Structural dynamics of synapses *in vivo* correlate with functional changes during experience-dependent plasticity in visual cortex. J Neurosci 2010; 30: 11086–11095. 20720116 10.1523/JNEUROSCI.1661-10.2010 PMC2932955

[bib27] Arime Y, Fukumura R, Miura I, Mekada K, Yoshiki A, Wakana S et al. Effects of background mutations and single nucleotide polymorphisms (SNPs) on the Disc1 L100P behavioral phenotype associated with schizophrenia in mice. Behav Brain Funct 2014; 10: 45. 25487992 10.1186/1744-9081-10-45 PMC4295473

[bib28] Shoji H, Toyama K, Takamiya Y, Wakana S, Gondo Y, Miyakawa T. Comprehensive behavioral analysis of ENU-induced Disc1-Q31L and -L100P mutant mice. BMC Res Notes 2012; 5: 108. 22348257 10.1186/1756-0500-5-108 PMC3392730

[bib29] Kvajo M, McLellar H, Drew LJ, Lepagnol-Bestel A,M, Xiao L, Levy RJ et al. Altered axonal targeting and short-term plasticity in the hippocampus of Disc1 mutant mice. Proc Natl, Acad Sci USA 2011; 108: E1349–E1358. 22049344 10.1073/pnas.1114113108 PMC3241761

[bib30] Frenkel MY, Bear MF. How monocular deprivation shifts ocular dominance in visual cortex of young mice. 2004 Neuron 44: 917–923. 15603735 10.1016/j.neuron.2004.12.003

[bib31] Johannessen M, Delghandi MP, Moens U. What turns CREB on? Cell Signal 2004; 16: 1211–1227. 15337521 10.1016/j.cellsig.2004.05.001

[bib32] Morris DW, Pearson RD, Cormican P, Kenny EM, O'Dushlaine CT, Perreault LP et al. An inherited duplication at the gene p21 Protein-Activated Kinase 7 (PAK7) is a risk factor for psychosis. Hum Mol Genet 2014; 23: 3316–3326. 24474471 10.1093/hmg/ddu025 PMC4030770

[bib33] Benito E, Barco A. CREB's control of intrinsic and synaptic plasticity: implications for CREB-dependent memory models. Trends Neurosci 2010; 33: 230–240. 20223527 10.1016/j.tins.2010.02.001

[bib34] Bennet MR. Schizophrenia: susceptibility genes, dendritic-spine pathology and gray matter loss. Prog Neurobiol 2011; 95: 274–300. 10.1016/j.pneurobio.2011.08.003 21907759

[bib35] Lepagnol-Bestel AM, Kvajo M, Karayiorgou M, Simonneau M, Gogos JA. A Disc1 mutation differentially affects neurites and spines in hippocampal and cortical neurons. Mol Cell Neurosci 2013; 54: 84–92. 23396153 10.1016/j.mcn.2013.01.006 PMC3637956

[bib36] Singh KK, De Rienzo G, Drane L, Mao Y, Flood Z, Madison J et al. Common DISC1 polymorphisms disrupt Wnt/GSK3β signaling and brain development. Neuron 2011; 72: 545–558. 22099458 10.1016/j.neuron.2011.09.030 PMC3387684

[bib37] Waltereit R, Weller M. Signaling from cAMP/PKA to MAPK and synaptic plasticity. Mol Neurobiol 2003; 27: 99–106. 12668903 10.1385/MN:27:1:99

[bib38] Wei J, Graziane NM, Wang H, Zhong P, Wang Q, Liu W et al. Regulation of *N*-methyl-D-aspartate receptors by Disrupted-in-Schizophrenia-1. Biol Psychiatry 2014; 75: 414–424. 23906531 10.1016/j.biopsych.2013.06.009 PMC3864617

[bib39] Wang Q, Charych EI, Pulito VL, Lee JB, Graziane NM, Crozier RA et al. The psychiatric disease risk factors DISC1 and TNIK interact to regulate synapse composition and function. Mol Psychiatry 2011; 16: 1006–1023. 20838393 10.1038/mp.2010.87 PMC3176992

[bib40] Hayashi-Takagi A, Takaki M, Graziane N, Seshadri S, Murdoch H, Dunlop AJ et al. Disrupted-in-Schizophrenia 1 (DISC1) regulates spines of the glutamate synapse via Rac1. Nat Neurosci 2010; 13: 327–332. 20139976 10.1038/nn.2487 PMC2846623

[bib41] Yoshii A, Constantine-Paton M. BDNF induces transport of PSD-95 to dendrites through PI3K-AKT signaling after NMDA receptor activation. Nat Neurosci 2007; 10: 702–711. 17515902 10.1038/nn1903

[bib42] El-Husseini AE, Schnell E, Chetkovich DM, Nicoll RA. PSD-95 involvement in maturation of excitatory synapses. Science 2000; 290: 1364–1368. 11082065

[bib43] Dawson N, Kurihara M, Thomson DM, Winchester CL, McVie A, Hedde JR et al. Altered functional brain network connectivity and glutamate system function in transgenic mice expressing truncated Disrupted-in-Schizophrenia 1. Transl Psychiatry 2015; 5: e569. 25989143 10.1038/tp.2015.60 PMC4471291

[bib44] Marder E, Goaillard JM. Variability, compensation and homeostasis in neuron and network function. Nat Rev Neurosci 2006; 7: 563–574. 16791145 10.1038/nrn1949

[bib45] Javitt DC. Neurophysiological models for new treatment development in schizophrenia: early sensory approaches. Ann N Y Acad Sci 2015; 1344: 92–104. 25721890 10.1111/nyas.12689 PMC4467888

[bib46] Dölen G, Osterweil E, Rao BS, Smith GB, Auerbach BD, Chattarji S et al. Correction of fragile X syndrome in mice. Neuron 2007; 56: 955–962. 18093519 10.1016/j.neuron.2007.12.001 PMC2199268

[bib47] Tropea D, Giacometti E, Wilson NR, Beard C, McCurry C, Fu DD et al. Partial reversal of Rett Syndrome-like symptoms in MeCP2 mutant mice. Proc Natl Acad Sci USA 2009; 10: 2029–2034. 10.1073/pnas.0812394106 PMC2644158 19208815

[bib48] Booth CA, Brown JT, Randall AD. Neurophysiological modification of CA1 pyramidal neurons in a transgenic mouse expressing a truncated form of disrupted-in-schizophrenia1. Eur J Neurosci 2013; 39: 1074–1109. 10.1111/ejn.12549 PMC4232873 24712988

