# Supplementary material for: Disrupted in schizophrenia 1 (DISC1) L100P mutants have impaired activity-dependent plasticity in vivo and in vitro
Source: Transl Psychiatry. 2016 Jan 12;6(1):e712–. doi: 10.1038/tp.2015.206 (PMC5068880; doi:10.1038/tp.2015.206)
Supplement: Supplementary Information [file tp2015206x1.docx]

**Tropea et al., Supplementary Information**

**Materials and Methods**

**DNA extraction and genotyping**. In order to determine mice genotype (wild type, heterozygous or homozygous for the L100P mutation), allelic discrimination assay was performed by real time-PCR starting from mice tail tissue. DNA was extracted from each tail using Wizard SV Genomic DNA Purification System (Promega, A2361). Tissue samples were singularly placed in 0.5 ml labelled tubes; approximately 1.5 cm of tail tissue was immersed in 275µl of lysis buffer containing 200µl Nuclei Lysis Solution, 50µl 0.5M EDTA pH=8, 20µl Proteinase K (Promega, V3021) and 5µl RNase solution. Samples were then incubated for 18 hours at 55°C using a PCR thermocycler. DNA isolation from lysed tissue samples was performed at RT (room temperature) according to the manufacturer instructions and consisted on a few steps of samples filtering and washing, followed by DNA elution with nuclease free water. DNA concentration for each sample was determined by spectrophotometric reading (Nanodrop, ND8000). DNA samples were stored at -20°C. Genotypes were established by multiplexed real time-PCR performed using the Applied Biosystems 7900HT sequence detector (SDS 2.3 software): a primers/probe specific for the wild type allele and a primers/probe specific for the mutant allele, which target the SNP (single nucleotide polymorphism) site, were used in each reaction. Samples were loaded in triple into 96-well plates and contained: 10ng DNA, TaqMan Universal Master Mix no AmpErase UNG (Applied Biosystems, 4324018), and a premade TaqMan assay primers/probes mix purchased from Applied Biosystems. The primers/probes mix contained forward/reverse *Disc1* primers (*Disc1*_F: CCA CTG CCA AGC CTC ACT; *Disc1*_R: GGC CAC AGC AGG GAC AA) and genotype-specific MGB (minor groove binder) probes, which were conjugated to different reporter dyes (*Disc1* wild type allele: AGC TCT TGA GAA AAG, VIC-conjugated; L100P mutant allele: AGC TCT TGG GAA AAG, 6-FAM-conjugated). Nuclease free water was used as a negative control and genotypes were established by evaluation of the amplification curves generated by the machine and plotted as reporter fluorescence against PCR cycle. Only homozygous wild type and L100P mutant animals were selected for the experiments described.

**Primary Neuronal Cultures.** Primary neuronal cultures were made from brain cortices of DISC1 L100P mice and WT littermates at postnatal day 0 (P0) or P1. Animals were euthanatized by decapitation, brains were dissected right after in ice-cold sterile DPBS (Dulbecco’s phosphate buffered saline, gibco, 14190-094) and cortex was finely minced in 1mm^3^ pieces. Tissue was moved to sterile tubes in a volume of 200 µl DPBS and added with 2 units DNase (DNase I RNase-free, New England BioLabs, M0303S) and 20µl trypsin-EDTA (solution of 0.025%trypsin and 0.01%EDTA in PBS, gibco, R-001-100) for cell dissociation. Tubes were incubated at 37°C and 5%CO_2_ for 10 minutes, enzymatic reaction was then blocked by adding a double volume of DMEM media (Dulbecco’s modified Eagle’s medium-high glucose, Sigma, D5796) containing 10% FBS (foetal bovine serum GOLD, PAA, A15-101). Cells were mechanically dissociated by gentle pipetting and kept at room temperature to settle down for 5-15 minutes. Supernatant was collected from each sample and moved to clean tubes that were spun for 5 minutes at 10°C and 800rpm to pellet dissociated cortical cells. Supernatant was this time discharged; pelleted cells were resuspended in 10%FBS/DMEM and counted using a haemocytometer under a light microscope with 10X magnification. Cells were plated at the density of 1X10^6^ cells/ml, into 24well plates onto glass coverslips, previously coated with poly-L-lysine (Sigma, P4707). Plates were kept at 37°C/5%CO_2_ and fed 2 hours later with feeding medium (FM) to achieve a volume of 500µl/well. Feeding medium contained: 95% neurobasal medium (neurobasal medium [-]-L-glutamine, gibco, 21103-049) containing 1% P/S (penicillin/streptomycin, 50 units/ml, gibco, 21103-049), 2mM L-glutamine (gibco, 15140-163), 0.45% glucose (D-(+)-glucose solution 45%, Sigma, G8769) and 2% B27 (50X B27 serum-free supplement, gibco, 17504-044). After 24 hours incubation at 37°C and 5%CO_2_, cells were fed by replacing half FM volume with fresh FM containing 7µM AraC (cytosine β-D-arabinofuranoside, Sigma, C1768) in order to achieve a final concentration of 3.5µM AraC in each well. Cytosine β-D-arabinofuranoside or AraC is a mitotic inhibitor, which is used at low concentrations as an inhibitor of glial growth. Cells were kept at 37°C and 5% CO_2_, checked using a light microscope and fed every second day by replacing half medium with fresh FM containing 3.5µM AraC. Cells were grown as above until 6 days in vitro (6DIV), when they were treated as described in detail below. At 8DIV cells were fixed for 20 minutes using a PBS solution containing 4%PFA (paraformaldehyde), washed with PBS and kept at 4°C until immunostaining experiment were carried out.

**Chemical treatment of primary neuronal cultures.** Protocol for chemical induction of LTP (long-term potentiation) was adapted from (Roth-Alpermann et al. 2006), LTP mix contained- was applied for 10 minutes and prepared as follows: 124mM NaCl (sodium chloride), 5mM KCl (potassium chloride), 1.25mM KH_2_PO_4_ (monopotassium phosphate), 5mM CaCl_2_ (calcium chloride), 24mM NaHCO_3_ (sodium bicarbonate), 25mM TEA (tetra-ethyl-ammonium chloride), 10mM D-glucose; pH was adjusted at 7.2 using 1M HCl solution, the mix was filtered in sterile conditions and warmed up to 37°C before use. In all experiments, control cultures treated with vehicle solutions were included.

**Immunocitochemistry** All immunostaining steps were performed at room temperature; plates underwent gentle shacking all the time except during primary antibody incubation, when plates were kept on the lab counter motionless. After a few washes with PBS, cultures were incubated for 1 hour in a solution made in PBS containing: 10% goat serum for aspecific binding sites-blocking, and 0.1% triton X-100 for cell permeabilization. Cultures were then incubated overnight with primary antibody solution, which was made as the blocking solution above and added with two or three different primary antibodies. After about 16 hours, cultures were rinsed 3 times with PBS solution for a total time of 15 minutes in order to remove unbound primary antibody, and incubated for 2 hours with secondary antibodies diluted in a solution of 0.05%triton X-100/PBS. Secondary antibodies used were conjugated to fluorescent dyes; plates were kept covered by thin foil from this step onward in order to avoid fluorescence decay. Cultures were finally washed with PBS and coverslips were mounted onto glass microscope slides using Vectashield Hard Set with DAPI (Vector, H-1500). A “no primary/secondary antibodies only” control was performed for each experiment, in order to visualize possible aspecific signal given by cellular autofluorescence or by the background. Cortical cultures were made from at least three different animals for chemical treatments experiments. For each animal/treatment one or two coverslips were stained, and at least 10 fields were imaged from each coverslip. Culture samples were imaged using a Zeiss confocal microscope with 63X oil immersion-objective using non-saturing settings. Only images showing neurons clearly distinguishable from the background staining were carried forward for analysis. Images were analyzed using ImageJ as follows. For the experiment involving chemical treatments, area (or length) and integrated density were measured for each neuron included in the analysis. The integrated density values were used as a measure of the immunostaining (IS). Background fluorescence was measured and subtracted from cellular IS signal. For each experimental replicate, the average IS intensity of each experimental group was normalized to the average IS intensity of the control treatment. Resulting measures of each experimental replicate were now expressed in control IS-fold, which allowed us to pool together results from several independent replicates. Since not all the distributions had a normal distribution, differences among treatments effects were assessed with the Mann-Withney test.

In order to measure dendritic length a segmented line was drawn along each primary dendrite and its length was measured. The integrated density of synaptic proteins was measured on the same dendrite, so the IS is reported as integrated density/length. For nuclear staining, the nuclear area was calculated and the integrated density in the nucleus measured. The IS is reported as integrated density/area.

**Immunohistochemistry**. Mice were perfused with a peristaltic pump at a speed of 1 pulse/second. First, cold saline solution and then cold 4% paraformaldehyde (PFA) were used to fix the tissues. Brain was extracted and post-fixed in cold PFA for 2 hours, and then moved to 20% sucrose for cryoprotection. Brains were sliced in coronal sections of 50 µs for immunostaining. All protocol steps were carried out in gentle agitation at RT unless otherwise specified. Between one step to another, sections were gently moved from a well to another in the plate using a glass hooked Pasteur pipette. Sections were incubated in fresh made hydrogen peroxidase solution (0.1% H_2_O_2_/PBS) for 5 minutes. Tissue was washed 4 times in PBS for a total time of 20 minutes and incubated for 1 hour in a solution of PBS containing 10% goat serum (Sigma, G9023) and 0.3% triton X-100 (Sigma, T8787). Triton X-100 was used to achieve tissue permeabilization for proper antibody penetration, and goat serum was used for covering aspecific binding sites. Sections were then probed overnight with two or three primary antibodies specific for different antigens, which were diluted in a PBS buffer containing 1% goat serum and 0.3% triton X-100: this step was performed at 4°C, in static conditions, and lasted approximately 48 hours in order to guarantee optimal primary antibody binding throughout the thickness of the tissue. The excess of primary antibody was washed off with several changes of PBS for a total time of 2 hours. Washes were followed by incubation with secondary antibody mix, in which fluorophores-conjugated immunoglobulins were diluted in 1%goat serum/0.1%triton X-100/PBS for 2 hours: during this step, plate was covered with thin foil in order to preserve fluorescence. Fluorophores used included DyLight488 (green light emission), Cy3 (red light emission) DyLight649 (far red/purple light emission). For pCREB staining, which required signal amplification, a secondary antibody conjugated to biotin was used, followed by 1 hour incubation with ExtrAvidin-FITC conjugated. Fluorescein isothiocyanate (FITC) is a fluorophore that emits green light and can be used in alternative of DyLight488. Tissue sections were finally washed 3 times for 10 minutes with PBS, and mounted onto glass microscope slices with the help of a small paintbrush, and using non-hardening mounting medium containing DAPI (Vector, H-1200). DAPI (4',6-diamidino-2-phenylindole) is a blue fluorescent dye with strong affinity for DNA: it was used to stain cellular nuclei. Slides were protected from light and kept at 4°C until they were imaged.

Image analysis. Imaging of the stained sections was performed by confocal microscope (Zeiss). Visual Cortex was identified on each brain section and for each field of view, a variable number of Z stacks were imaged through the thickness of the tissue, with a distance of 5µm between two consecutive stacks; from the majority of the fields four consecutive stacks were imaged, and no more than five.

In all cases, a Zeiss 63X magnification oil-immersion objective (numerical aperture/NA = 1.4) was used for confocal microscope imaging. In order to avoid image saturation and to assure unbiased image detection across experimental groups in all experiments, the following procedures were performed:

1. All the experimental groups for each experiments were imaged during the same session to avoid bias due to the intrinsic variability of the microscope (i.e. time of laser operativity).
2. Sampling imaging for each experimental group was used to set up the superior and inferior thresholds of the parameters. The parameters considered were: laser intensity, pinhole, detection gain, offset. Laser intensity and pinhole were kept constant throughout each imaging sessions. In order to set amplification gain and offset we used the HiLow scale, which shows the level of saturation and understimulation for each pixel. Attention was paid that the pixel in the images were not saturated or understimulated. Once conditions were set for one group, sample imaging was done on the other experimental groups to ensure that the conditions set were in the right range also for the other groups. Once the parameters were set, they were kept constant for all the images of each and every experimental group.

As in all procedures of immunodetection, when signal is not saturated, intensity is directly proportional to the amount of antigen in the sample. Measurement of immunostaining (IS) intensity was carried out using ImageJ and following different criteria of analysis, depending on brain area analyzed and subcellular localization of the antigen of interest. For quantification of IS intensity, single cells or broader tissue surface were selected: the sum of the value of bright pixels in the selected surface (or integrated density) was normalized to the measure of the area. Also, three surfaces of background were measured with the same criteria and average value was calculated. For each field, normalized background value was subtracted from normalized IS value, so as to obtain an IS value net of background. In all cases differences between groups were statistically assessed by Mann-Whitney test, and considered significant when P value was ≤0.05.

Primary antibodies used: (synapsin SYSY 106004 - 1:500; PSD95: Cell Signaling 2507 – 1:500; PCREB: Cell Signaling 9191- 1:300, MAP2: Millipore MAB3418). Secondary antibodies used: Cy3-conj GT anti GP: JIR106-165-003; DL488-conj GT ant RB JIR 111-485-003; GT anti RB biotinilated VECTOR; DL649-gonj GT anti MS JIR115-495-003.

**Monocular Deprivation:** Monocular deprivation was performed during the critical period on anesthetized mice (avertin, i.p.,) in sterile conditions. Under a surgical microscope, the upper and lower eyelids of one eye were sutured closed in register with closely spaced sutures. Animals are allowed to revive with supplemental heating under close supervision, and are returned to the home nest when fully revived. Sutures were removed after 5 days right before the imaging session.

**Optical Imaging of intrinsic signals:** Once sufficiently anesthetized (urethane, 1.5 gr/Kg), the head of the animal was fixed in a stereotaxic frame and the skull exposed. Using a dremel drill, the skull was thinned over V1 and V2. A chamber was placed on the region of interest and covered with a coverslip. During the imaging sections, the cortical surface was illuminated with a tungsten halogen light source and images of intrinsic signals were acquired with a slow scan video camera equipped with a tandem macro-lens arrangement. First the cortical surface was illuminated with a green filter (550nm) to obtain a reference image of the surface blood vessels. Then, the focal plane was adjusted to about 300 microns below the cortical surface and a red filter (630nm) was used for the acquisition of intrinsic signal maps. To obtain ocular dominance maps, a drifting bar (4 orientations, spatial frequency of 9 deg, temporal frequency of 0.1 Hz) was presented to each eye individually. The monocular responses to the same orientation (elevation) were pooled and a comparison made between the two eyes. During the whole procedure the animal received oxygen and its conditions were monitored.

**Visual Cliff test:** the test was performed on adult mice and their whiskers were trimmed to prevent the sensory detection of the platform, and assure that the only criteria to move to one side of the box was dependent on visual abilities. Mice were placed in a 60×60 cm box with a clear Perspex base. A high-contrast grating was attached to the underside of one half of the box; the clear half of the box was placed such that it protruded from the table, revealing a drop to the floor of approximately 90 cm. Between the two halves we positioned an elevated platform (6 x 16x 1.5 cm) Mice were placed in the centre of the platform and their behaviour was monitored. In particular, we evaluated whether the mouse showed preference for the patterned side or the other side. Particular care was taken to avoid that the reflection at the bottom of the platform would affect the test.

**Statistical analysis:** For statistical analysis, we used “Excel-analyze it”, and non –parametric comparison Mann-Whitney. Statistical differences were considered significant for a p <0.05.

**Reference**

Roth-Alpermann, C., Morris, R.G., Korte, M., and Bonhoeffer, T. (2006). Homeostatic shutdown of long-term potentiation in the adult hippocampus. Proc. Natl. Acad. Sci USA 103, 11039-11044.
