# Supplementary figures and images for: Disrupted in schizophrenia 1 (DISC1) L100P mutants have impaired activity-dependent plasticity in vivo and in vitro
Source: Transl Psychiatry. 2016 Jan 12;6(1):e712–. doi: 10.1038/tp.2015.206 (PMC5068880; doi:10.1038/tp.2015.206)

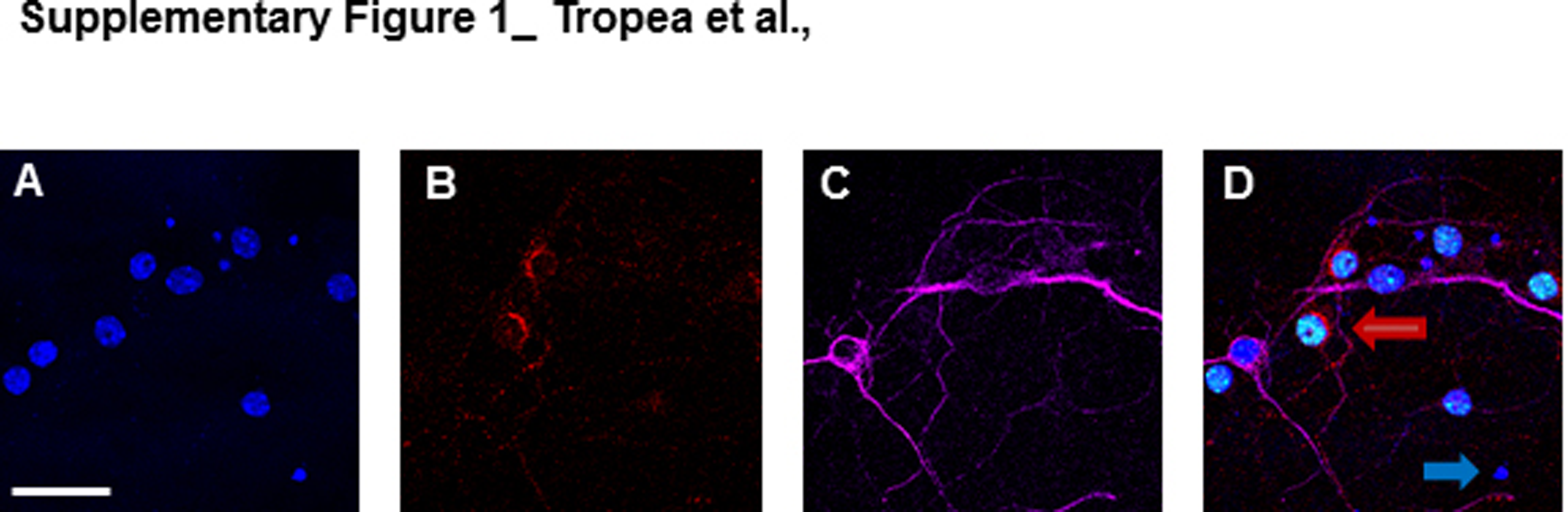

Supplement: Supplementary Figure 1 [file tp2015206x2.tif]

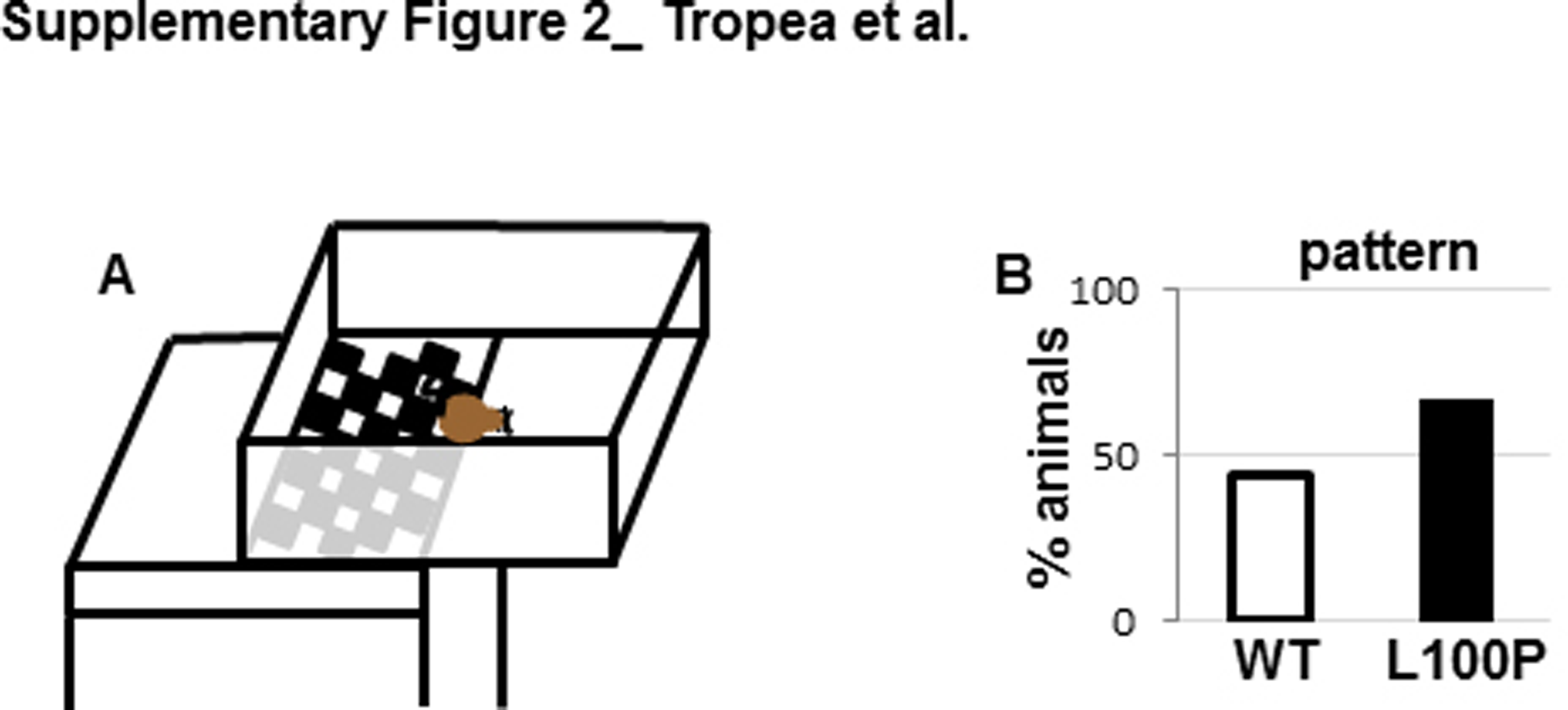

Supplement: Supplementary Figure 2 [file tp2015206x3.tif]

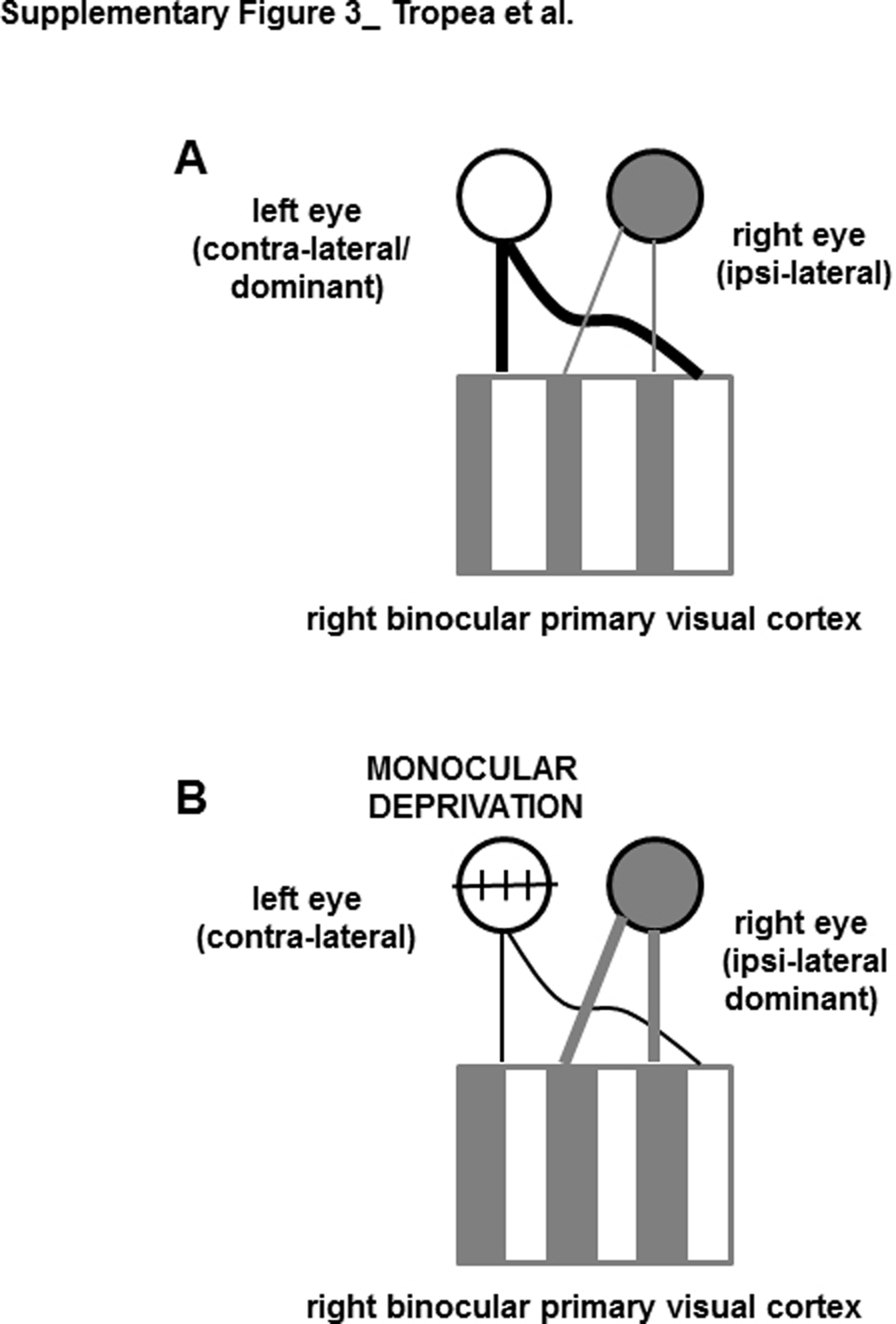

Supplement: Supplementary Figure 3 [file tp2015206x4.tif]

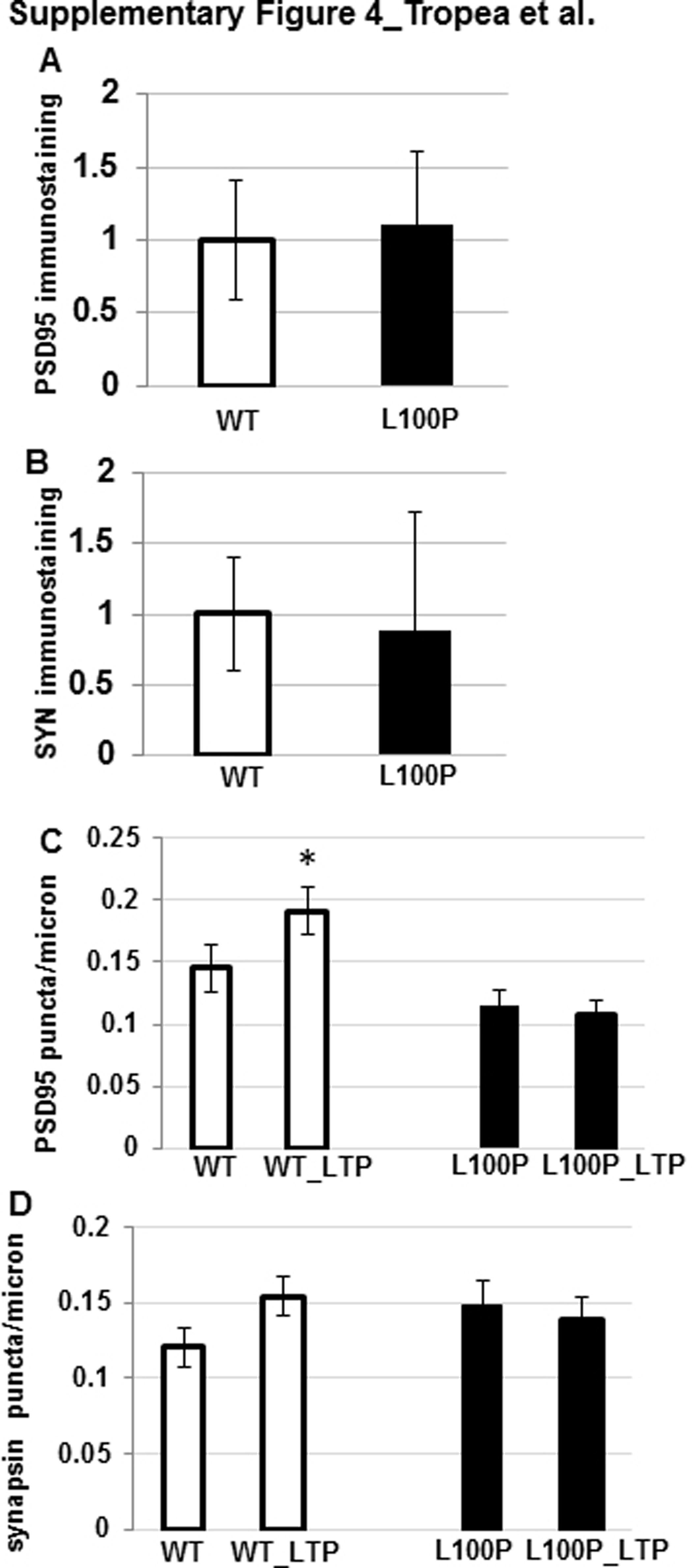

Supplement: Supplementary Figure 4 [file tp2015206x5.tif]

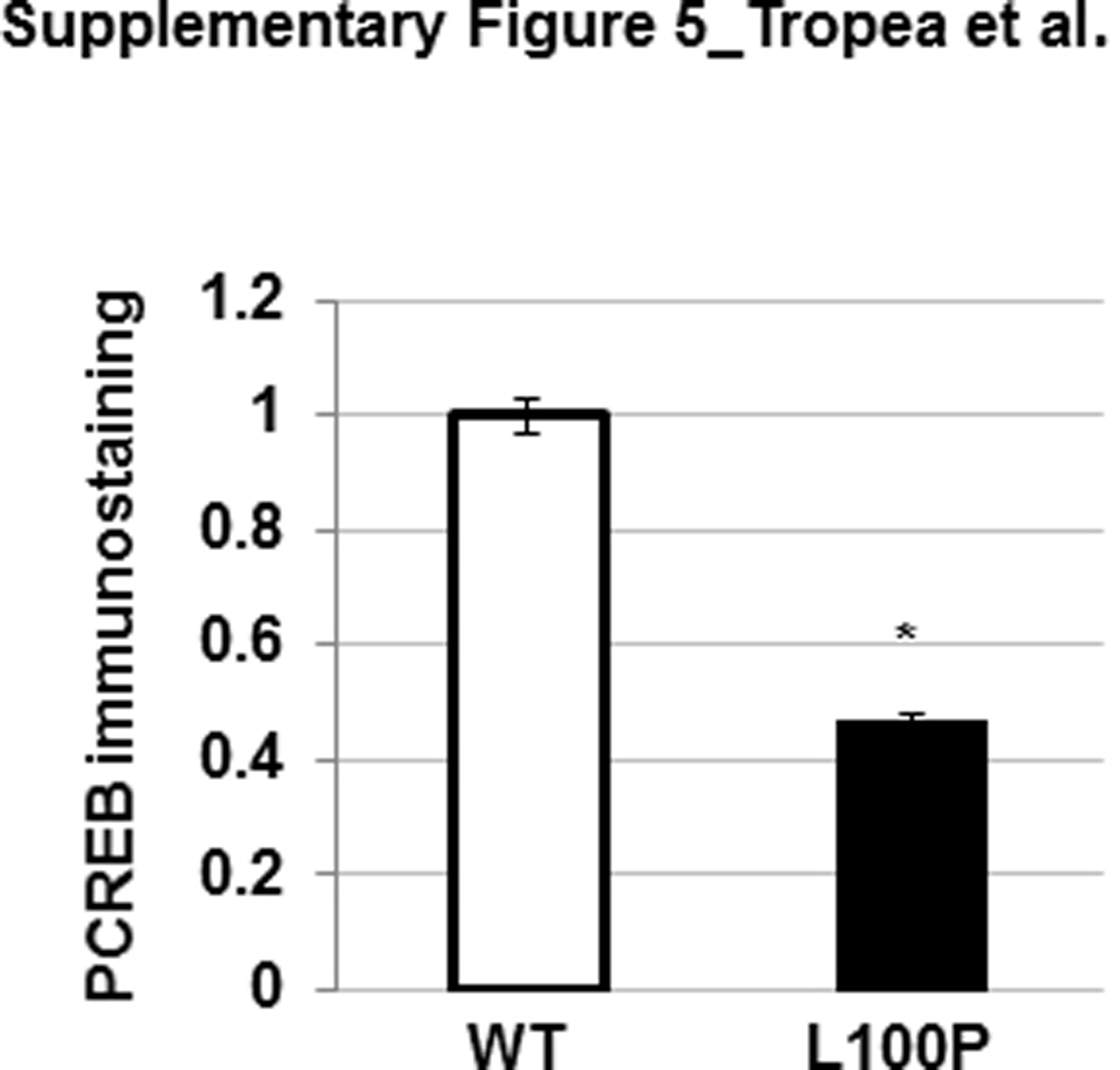

Supplement: Supplementary Figure 5 [file tp2015206x6.tif]

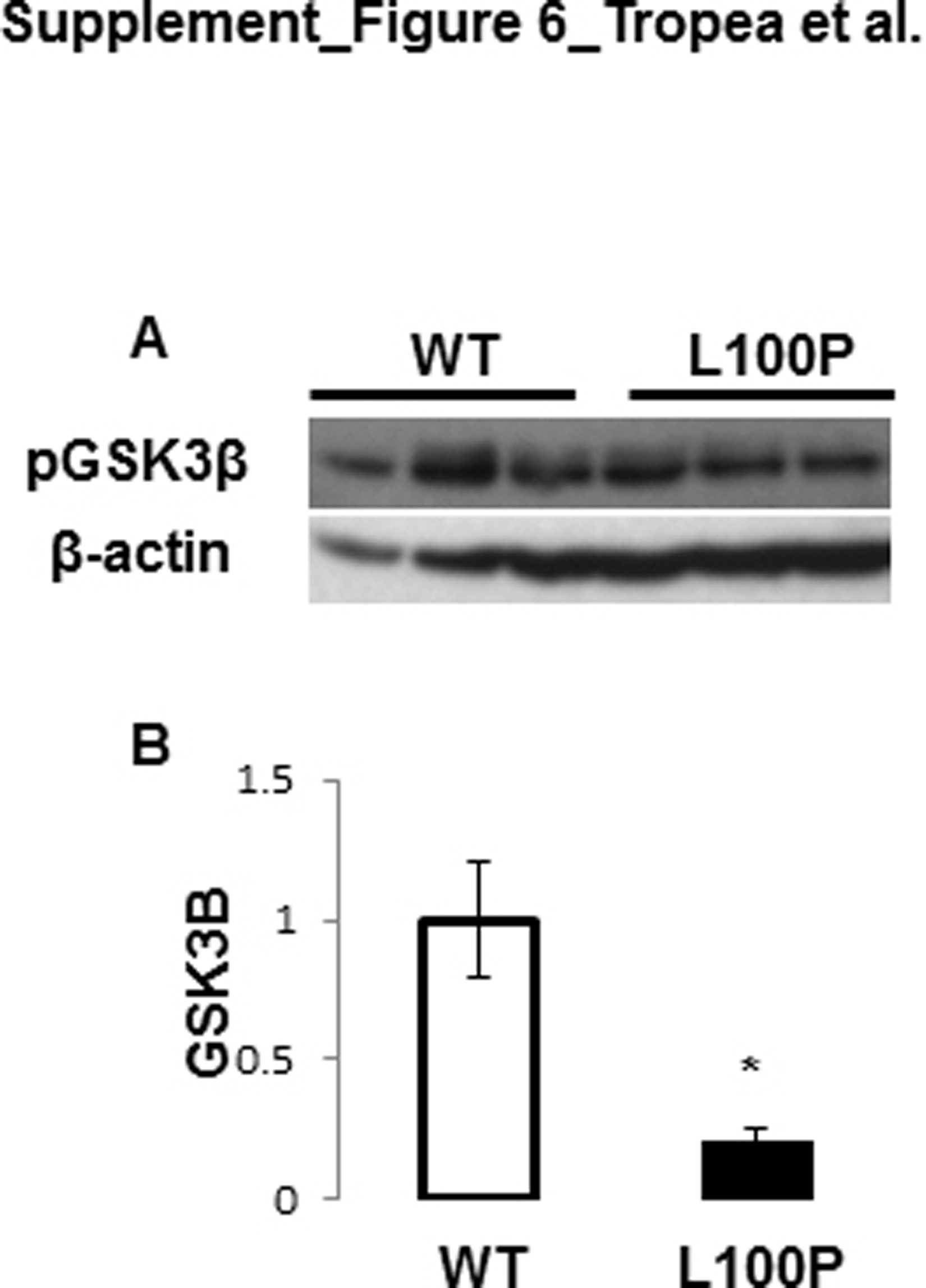

Supplement: Supplementary Figure 6 [file tp2015206x7.tif]

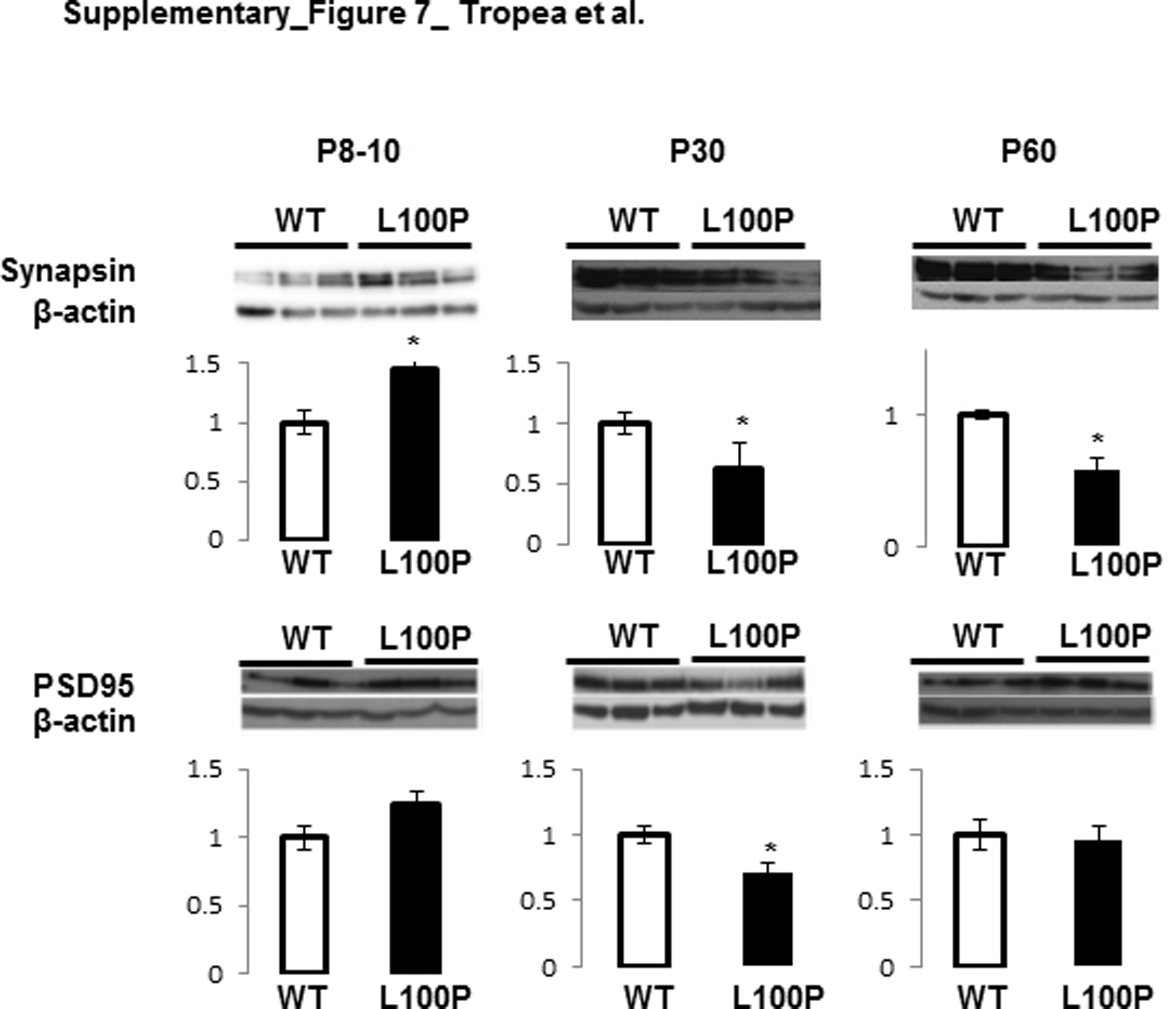

Supplement: Supplementary Figure 7 [file tp2015206x8.tif]

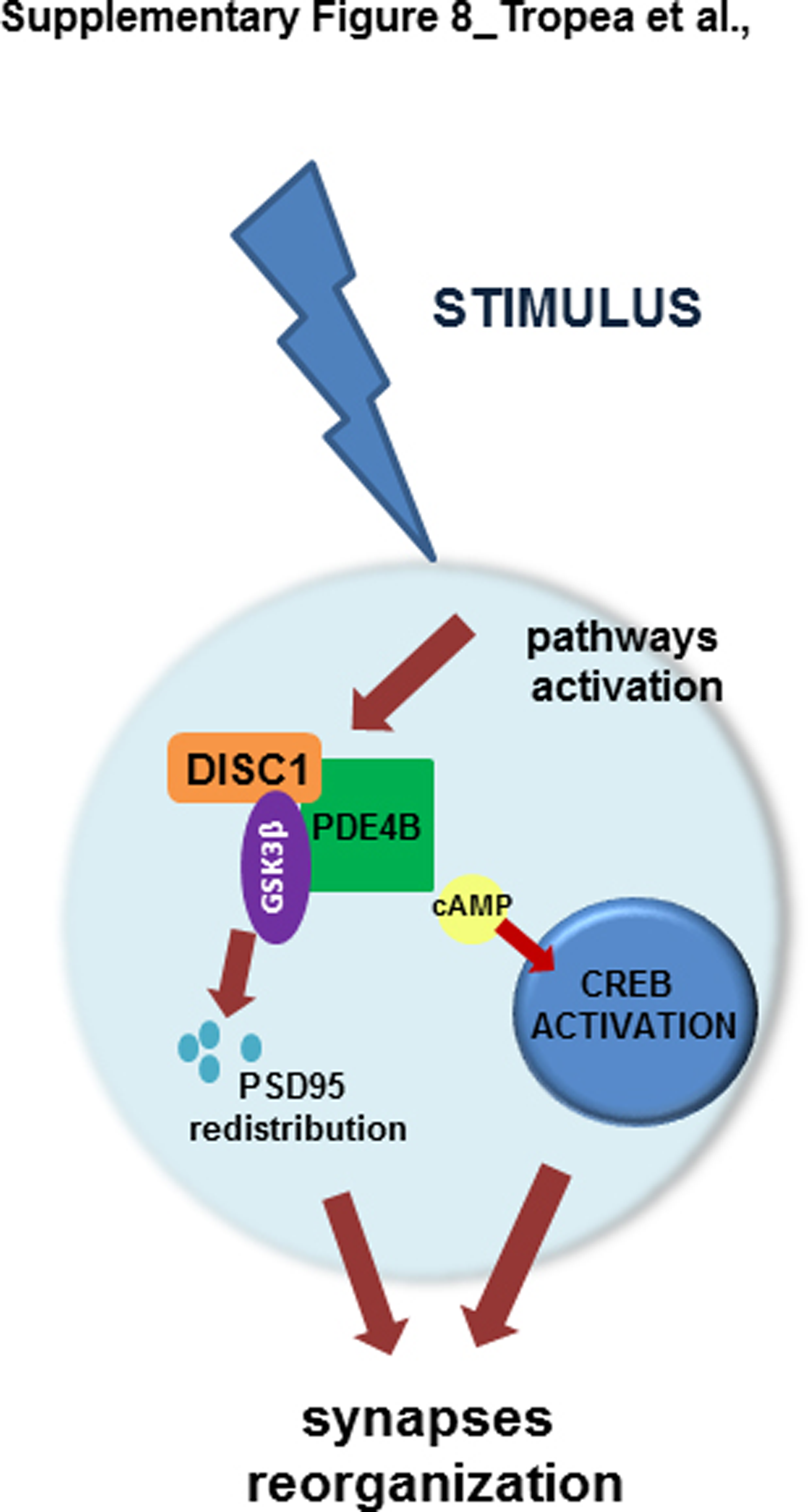

Supplement: Supplementary Figure 8 [file tp2015206x9.tif]
